# A Plasmonic Fiber Based Glucometer and Its Temperature Dependence

**DOI:** 10.3390/mi9100506

**Published:** 2018-10-05

**Authors:** Jisoo Kim, Changjin Son, Sunjak Choi, Won Jung Yoon, Heongkyu Ju

**Affiliations:** 1 Department of Nano-Physics, Gachon University 1342 Seongnam-daero, Sujeong-gu, Seongnam-si, Gyeonggi-do 13120, Korea; wltndi95@naver.com (J.K.); watchmaker@nate.com (C.S.); sunjakchoi@naver.com (S.C.); 2 Gachon Bionano Research Institute, Gachon University 1342 Seongnam-daero, Sujeong-gu, Seongnam-si, Gyeonggi-do 13120, Korea; 3 Department of Chemical and Bio Engineering, Gachon University 1342 Seongnam-daero, Sujeong-gu, Seongnam-si, Gyeonggi-do 13120, Korea; wjyoon@gachon.ac.kr; 4 Neuroscience Institute, Gil Hospital, Incheon 405-760, Korea

**Keywords:** fiber SPR, biosensor, optical glucometer, glucose, temperature stability, enzyme electrode

## Abstract

We present the plasmonic fiber based optical glucometer. A thin gold layer is coated on clad-free core of multimode optical fiber along 3 cm length to excite surface plasmons at 632.8 nm wavelength. Glucose oxidase is immobilized on the metal surface for glucose sensing. The effective surface refractive index increases by gluconic acid and hydrogen peroxide that are generated upon glucose injection, leading to plasmonic condition change with a consequence of optical power change at the fiber output. We obtain limit of detection of glucose concentration of 6.75 mg/dL, indicating higher sensitivity than the wavelength interrogating SPR glucometer that uses a spectrometer of 1nm spectral resolution. The coefficient of variation is 8.6% at a glucose concentration of 80 mg/dL at room temperature. We also examine the effects of ambient temperature variations from −10 °C to 40 °C on the performance of the presented sensor and compared them with those on commercially available glucometers that are based on enzyme electrodes. We find that the presented fiber sensor produced standard deviation of 12.1 mg/dL at a glucose concentration of 80 mg/dL under such varying temperature, which is, even without additional temperature correction function, comparable to the commercialized ones.

## 1. Introduction

Blood glucose monitoring has been increasingly important for controlling progress of disease, including diabetes [1]. Demand for blood glucose self-monitoring has driven the development of various types of glucose measuring devices, such as enzyme electrode based electrochemical sensors [2,3,4,5], colorimetric sensors [6,7,8,9,10,11], and optical devices [12,13,14,15,16] that measure glucose levels. Optical fibers have been used for glucose sensing to harvest its advantages of remote sensing capabilities and relatively simple/tiny format of the sensor platform. They been demonstrated by means of the measurement of optical spectrum changes (peak wavelength shift) induced by refractive index changes due to glucose oxidation [17,18]. In the fiber based glucometers, the glucose oxidation occurs via gel entrapped glucose oxidase on sensing surface.

Requirement for accurate measurement of glucose level is of great importance for credible quantitation of glucose level. Among a number of factors affecting glucometer performance, such as ambient temperature, humidity, and atmospheric pressure, ambient temperature can be considered one of the most important external disturbances that influence glucose level measurement. This is particularly true of conventional glucometers, i.e., enzyme electrode based glucometers that rely on efficient transport of glucose oxidation generated electrons, which would be sensitively subject to ambient temperature variation. It is also seen that gel entrapment of glucose oxidase demonstrated in the previous glucometers, such as those based on fibers, might be subject to its possible volume change caused by ambient temperature variation, i.e., expansion or contraction.

In this paper, we present an optical fiber based device as an alternative glucometer that enables glucose level to be measured quantitatively with glucose oxidase chemically bonded on its sensing surface (without gel entrapment), and investigate dependence of the measured glucose level on ambient temperature. We use surface plasmon resonance (SPR) coupled with fiber optical modes for glucose level measurement. SPR can be excited by coating a thin gold (Au) layer on core of an optical multimode fiber that is stripped of its plastic cladding along 3 cm length, forming a sensor head. Optical modes propagating along a multimode fiber enables SPR to be excited in the sensor head. SPR condition changes as the surface effective refractive index on the metal surface changes [19,20,21,22,23,24,25], leading to optical power change at the fiber output. Unlike the previous works [17,18], this power measurement scheme for glucose detection enables us to avoid use of a spectrograph whose size becomes increased for higher precision. We immobilize the glucose oxidase (GOx) on its surface, enabling the surface effective index to change upon glucose introduction as a result of glucose oxidation that produces the by-products of higher indices, i.e., gluconic acid and hydrogen peroxide (H_2_O_2_). We use the glucose concentration ranging from 0 to 200 mg/dL for the experiment and obtain limit of detection of 6.75 mg/dL and coefficient of variation of 8.6% at glucose concentration of 80 mg/dL, the sensitivity being much lower than a SPR optical glucometer that employs a spectrometer of 1 nm spectral resolution.

We also investigate the dependence of estimated glucose levels on ambient temperature ranging from −10 to 40 °C at 80 mg/dL glucose concentration, and compare it with the commercialized glucometer that use enzyme electrodes. From room temperature to just below 40 °C, the presented sensor produces a quite stable estimation of the glucose level. Under the whole temperature variation, the presented fiber sensor produces the standard deviation of 12.1 mg/dL. This is comparable to the two commercialized glucometers, even without using the additional function of temperature correction in the presented fiber sensor.

## 2. Experimental Techniques and Materials

### 2.1. Materials and Instruments

Ethylene glycol (324558-100ML), D(+)-Glucose (G8270-100G), Glucose Oxidase from Aspergillus niger (G2133-10KU), Sodium(meta) periodate (71859-25G), and Cysteamine hydrochloride (M6500-25G) were purchased from Sigma-Aldrich Co. (St Louis, MO, USA). A sylgard 184 elastomer kit composed of silicone elastomer base and silicone elastomer curing agent (Dow corning, Midland, MI, USA) was used to make Polydimethylsiloxane (PDMS) flow cell. A peristaltic pump (SMP-21, Eyela Co. Ltd., Shanghai, China) was used to drive liquid flow through tubings that were connected to the inlet and outlet of the flow cell. Silica polymer clad optical fiber (JTFLH200230500) (200 μm core diameter, 230 μm clad diameter and 0.37 numerical aperture) was bought from Polymicro Technologies (Molex, Lincolnshire, IL, USA). A temperature controller was purchased from HTRD (Anyang, Korea) to control the temperature inside a chamber where a flow cell and fiber sensor head were installed. A digital Abbe refractometer (DR-A1, ATAGO Co. Ltd., Tokyo, Japan) was used to check index of refraction of glucose solutions as control experiment. An optical power meter (1918-c, Newport Co., Newport Beach, CA, USA) was used to measure the optical power when investigating the temperature dependent performance of the plasmonic fiber sensor.

### 2.2. Solution Arrangement

We made sodium(meta) periodate solution with Deionized (DI) water of 1 mL and sodium meta periodate of 30 mg and mixed them with 10 μM GOx solution. The solution was stirred for an hour with a magnetic stirrer under 300 rpm in the dark. An hour later, glycol ethylene 6.97 μL was added to stop the reaction between GOx and sodium(meta) periodate [16]. The solution was again stirred for 30 min under 300 rpm and then filtered under 1000× *g* (centrifugal acceleration) for 10 min. DI-water of 700 μL was then added to the filtered solution (250 μL). We used this enriched GOx solution (330 μL) for GOx immobilization on each sensor head.

### 2.3. A SPR Fiber Sensor Head and Its Working Principle

**Figure 1:**
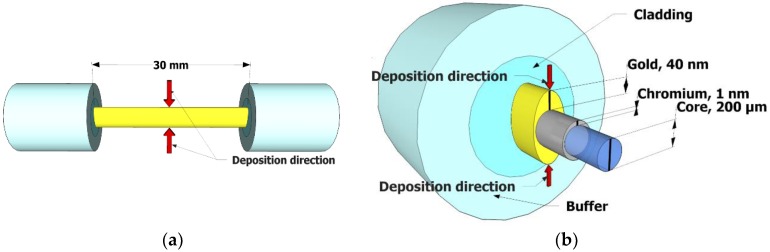
(**a**) A schematic of metal coated fiber. (**b**) The cross sectional profile of chromium and gold coated silica core of the fiber sensor head.

**Figure 2:**
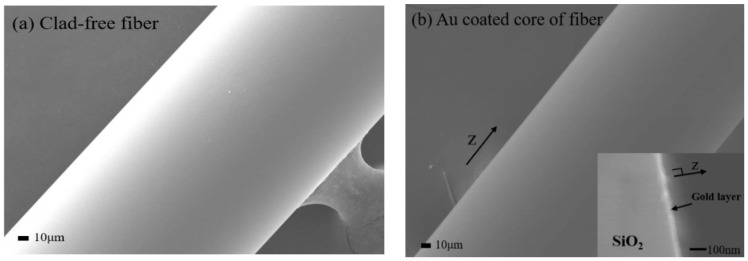
Scanning electron microscope (SEM) images for (**a**) clad-free fiber core and (**b**) Au coated core of the fiber (the inset image is a part of the fiber cross-section consisting of Au layer and silica core).

A multimode optical fiber was stripped off the plastic cladding along 3 cm length to expose its core [26,27,28]. Figure 1a shows schematic of the SPR fiber head. We chose to use Au for plasmonic metal coating, since we also needed to immobilize the glucose oxidase on the metal surface. As adhesion layers, we coated 1 nm-thick chromium (Cr) on one side of the fiber core and turned it over to coat the same metal on the other side. Then, we coated 40 nm-thick Au film on one side, turned it over, and repeated such Au coating on the other side. We would then expect an elliptical profile of the cross-section of the sensor head, as shown in Figure 1b. It was experimentally unveiled that coating of the thickness other than 40 nm of Au layer (for coating on both sides) would yield poorer sensitivity in the presented fiber sensor as long as the thermal evaporator system (Daeki Hi-Tech Co. Ltd., Daejeon, Korea) was used. The coating thickness of ≤40 nm around the core could support a wide range of incident angle of light at core-metal interface [29]. All of the metals were coated by a thermal evaporator with the pressure of 6 × 10^−6^ torr. Such elliptical profile of metal coating enabled various thickness, giving rise to the broadening of the plasmonic resonance condition. This broadening would also be supported by optical multimodes that propagate down the fiber at various incident angles with respect to the sensing surface. Figure 2a,b show the scanning electron microscope (SEM) images for a clad-free fiber core and Au coated fiber core. A part of the fiber cross-section comprises roughly homogenously coated Au layer and a silica core, as shown in the inset of Figure 2b.

**Figure 3:**
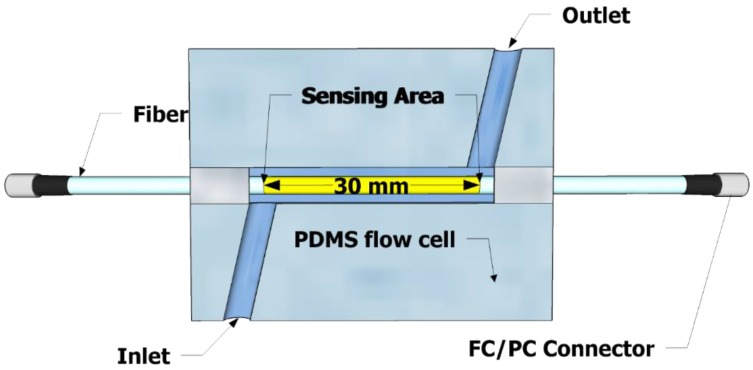
A top view of a Polydimethylsiloxane (PDMS) flow cell that allows liquid to flow and contact the sensor surface.

We fabricated a flow cell made out of PDMS which housed the plasmonic fiber sensor head, as shown in Figure 3, and allowed injected liquid to contact the sensor surface through an inlet and an outlet connected with plastic tubing driven by a peristaltic pump. The flow cell has the liquid filling volume of 350 μL.

**Figure 4:**
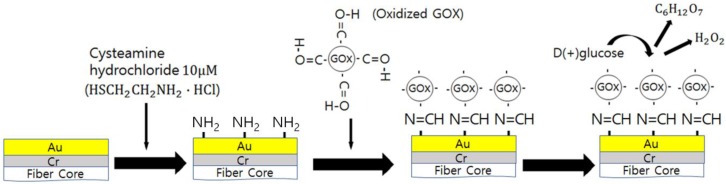
Sensor surface treatment for glucose sensing.

Figure 4 shows the step-by-step arrangement of the sensor surface for glucose sensing. We injected 10 μM cysteamine hydrochloride to the metal surface to generate amine groups with an incubation time of 90 min in the dark. The subsequent rinsing was performed with DI water to remove non-specific binding. We then immobilized oxidized GOx on the surface with an incubation time of 12 h in the dark and repeated the GOx immobilization process. This reaction was Schiff base reaction between the aldehyde group in oxidized GOx and amino group in cysteamine [30,31]. The subsequent glucose (C_6_H_12_O_6_) injection then produced gluconic acid (C_6_H_12_O_7_) and hydrogen peroxide (H_2_O_2_), as shown below

(1) $$C_{6}H_{12}O_{6}\;\left(\mathrm{glucose}\right)+O_{2}\overset{\mathrm{GOx}}{\to }C_{6}H_{12}O_{7}\;\left(\mathrm{gluconic}\;\mathrm{acid}\right)+H_{2}O_{2}\;\left(\mathrm{hydrogen}\;\mathrm{peroixde}\right)$$

The resultant products were the high index media (respective indices of 1.4161 and 1.414) that increased the effective surface index just above the plasmonic sensor surface. The enhancement of the effective surface index induces surface plasmons to be excited more resonantly, leading to more optical energies being dissipated. This gave rise to optical power (remainder of the optical power of light after optical energy consumption for surface plasmon excitation) decrease at the fiber output.

### 2.4. Experimental Setup

**Figure 5:**
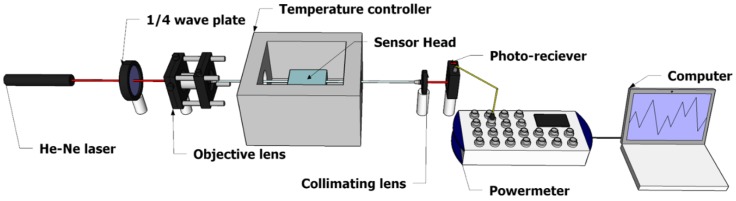
Experimental Setup for glucose sensing with the surface plasmon resonance (SPR) fiber sensor.

Figure 5 shows schematic of the experimental setup for sensing glucose with the plasmonic fiber sensor. We used a light source of a He-Ne laser at 632.8 nm wavelength. We followed it with a quarter wave-plate (λ/4), which produced a circular polarization to make sure half the optical power of light incident to the metal coated curved surface as p-polarized light. An objective lens was used to couple light into a multimode fiber that excited optical modes. The propagating optical multi-modes provided a wide range of ray incident angles to the metal surface, efficiently exciting SPR on such an elliptical profile of the coated metal cross-section (various metal thickness).

For glucose sensing, we injected glucose of various concentration (0 to 200 mg/dL) through a flow cell into the sensor head at room temperature. The higher concentration of glucose injected produced higher concentration of gluconic acid and hydrogen peroxide, resulting in higher effective index on the sensing surface. The surface plasmons derived more resonant condition from the higher index dielectric, thus more optical energies being consumed for plasmon excitation. This led to a decrease in fiber output power. The fiber output light that was collected by another lens was detected by a photo-receiver and recorded by a computer. A temperature controlling chamber that housed the sensor head and flow cell was used to control an ambient temperature under which the SPR fiber sensor was operated.

## 3. Results and Discussion

Refractive indices of glucose solution from 0 to 200 mg/dL were measured while using an Abbe refractometer. However, its limited index resolution of 10^−4^ RI could not distinguish the glucose solutions of different concentrations. To tell glucose concentrations apart and quantitate them further, we used the optical fiber sensor, which was expected to have the index resolution 10–100 fold better than an Abbe refractometer [32,33,34,35]. This could also be confirmed by the experiment with glycerol solution whose refractive index was known as a function of concentration.

**Figure 6:**
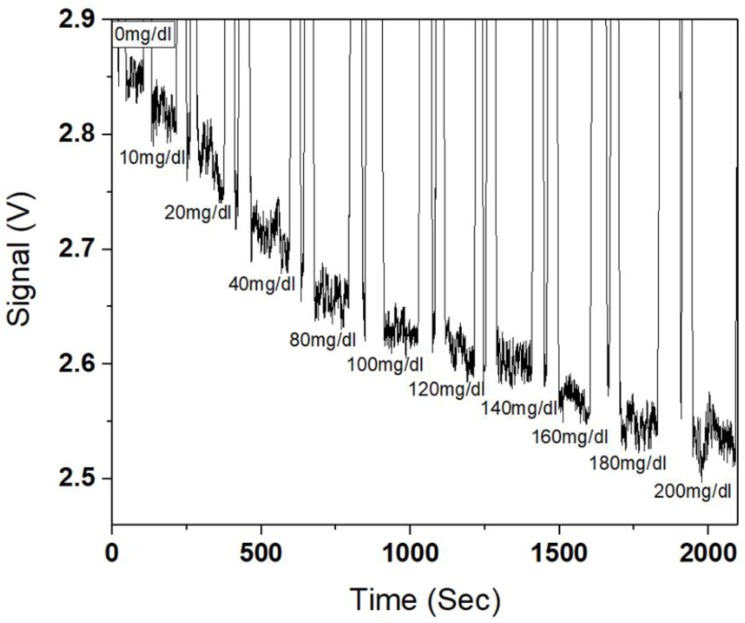
Plasmonic fiber output power (in volts) as a function of glucose concentration.

Figure 6 shows the real-time signal change as a function of glucose concentration of 0–200 mg/dL injected. The oxidation products upon glucose injection, i.e., C_6_H_12_O_7_ and H_2_O_2_ formed above the sensing surface, increased the effective surface index due to their higher refractive indices. The consequent change in fiber plasmonic resonance condition reduced the fiber output power, leading to monotonic power decrease with increasing concentration.

**Figure 7:**
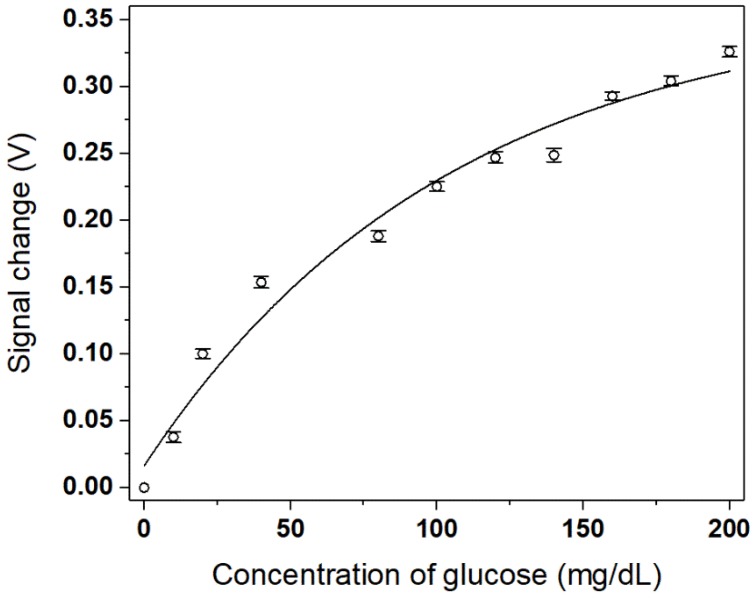
The magnitude of the sensor signal change as a function of glucose concentration. The solid line represents the exponential curve fitting to the data.

We repeated measurement at each concentration by rinsing the sensor surface with DI water. The magnitude in differential signal change averaged over repetition at each concentration is shown in Figure 7. We find that the sensor limit of detection (LOD) of glucose concentration against the three-fold standard deviation of a blank (zero concentration) sample signal, was estimated to be 6.75 mg/dL. (LOD was obtained while using a slope of signal vs concentration at near zero concentration and signal standard deviation at zero concentration. LOD was estimated as the concentration that corresponded to three times standard deviation of blank (zero concentration) signal). This appeared to be better than the previously demonstrated fiber based glucose sensors, such as the wavelength interrogating fiber SPR sensor device with a spectrometer of a resolution of about 1 nm [36] and the fiber terminal reflection based optical glucose sensor (14.2 mg/dL) [18]. The signal change magnitude tended to saturate with increasing concentration due to plasmonic evanescent field strength that decayed exponentially away from the sensing surface. Fitting to the data thus led us to use a kind of an exponential function, as given by

(2) $$y=a+be^{-kx}$$

where *y* is the signal change magnitude (in voltage) and *x* is the glucose concentration. a, b and *k* are the parameters used for fitting and may vary for different plasmonic metal coating and different concentrations of GOx immobilized on the metal surface. The parameter a, b, and *k* used in this fitting are 0.36186, −0.34557, 0.00962 respectively. The fitted curve permitted us to convert the measured signal to a glucose concentration. We used 80 mg/dL glucose to check the sensor reproducibility by repeating 10 times measurement at room temperature and obtain a coefficient of variation (CV) (equivalent of relative standard deviation in concentration estimated) of 8.6%. This CV was comparable or even better than the commercialized glucometers, even if no external disturbance factors, such as temperature and humidity, were taken into account for the final correction of concentration estimated. The plot and its fitting in Figure 7 also shows that the dynamic range for the glucose detection turned out to cover the range from zero to 200 mg/dL at least.

**Figure 8:**
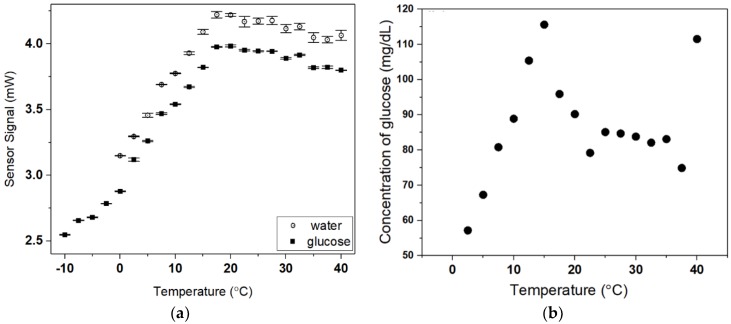
(**a**) Temperature dependent signal of the plasmonic fiber sensor at zero (DI water) and 80 mg/dL glucose concentration (**b**) The variation of concentration of glucose estimated by the sensor due to temperature variation.

We also investigated the dependence of ambient temperature on the presented sensor performance both at zero concentration (DI water) and at 80 mg/dL glucose concentration, as shown in Figure 8a. We chose to use 80 mg/dL glucose concentration for experiment since it was in the middle of the range of fasting blood glucose level of non-diabetic persons. We placed the sensor head in a chamber that could vary its internal temperature from −10 °C to 40 °C with a 2.5 °C step. At 80 mg/dL, the sensor signal (plasmonic fiber output power) increased with increasing the temperature from −10 °C to about 20 °C, as shown in Figure 8a. This is possibly due to reduced mass density of water solvent which caused the index to decrease. At zero concentration (water), the temperature dependent sensor signal showed behavior similar to that of 80 mg/dL, except for the fact that DI water being frozen at below 0 °C could not be measured. However, the signal decreased slightly at above 20 °C at both concentrations. This unexpected signal decrease at above 20 °C might arise from temperature dependent hardware variation, such as thermal expansion of the thin Au film. Overall, any kinds of temperature dependent effects on the sensor performance, except for pure glucose involved ones could be shared at both concentrations used, as seen in Figure 8a, and thus subtracted to derive pure glucose induced signal change. Therefore, we could take the DI water signal as a baseline and the signal difference between both concentrations for various temperatures enabled us to obtain temperature dependence of glucose concentration estimated while using the calibration curve presented in Equation (2).

It was seen that, at below room temperature, the plasmonic fiber glucometer produced non-negligible deviation from 80 mg/dL while producing fairly stabilized values of around 80 mg/dL over a limited temperature range from 22.5 °C to 37.5 °C. The standard deviation over the whole temperature range was 12.1 mg/dL. As shown in Figure 8b, glucose concentration estimated increased as the temperature rose from 2.5 °C to 15 °C. This could be due to more highly activated oxidation of glucose with an increasing temperature, and it thus produced more gluconic acid and hydrogen peroxide (higher refractive index effects), leading to an increase in glucose concentration estimated. At above 15 °C, the estimated concentration decreased possibly due to the reduced mass density of gluconic acid and hydrogen peroxide at increased temperature, considering higher likelihood (about 100 to 200-fold) of density reduction of such high index by-products than water [37]. This resulted in decreased refractive index, therefore counteracting the temperature effects that are mentioned above. The temperature dependence of glucose solubility could be ignored approximately due to small mass ratio of glucose less than 0.2% used in solution. We also checked the temperature dependence of glucose concentration estimated by two kinds of commercially available glucometers based on enzyme electrodes whereby electrochemical signals were used for glucose sensing. It was seen that those glucometers (which was believed to have additional function of temperature variation correction) showed glucose level standard deviation of 21.8 mg/dL and 8.8 mg/dL over the temperature variation.

## 4. Conclusions

We present the plasmonic optical fiber based glucometer whereby fiber multimode propagation excites surface plasmons, providing sensing mechanism under which the fiber output power reduces at higher glucose concentration. We coat a thin Au layer on the clad-free fiber core along 3 cm length for the plasmonic sensor head. This is followed by GOx immobilization on its metal surface by surface chemistry, differing from the gel entrapment technique, which is possibly vulnerable to temperature variation induced volume change. We demonstrate the LOD of glucose concentration of ~6.75 mg/dL, and CV of ~8.6% at 80 mg/dL. This sensitivity is much better than the wavelength interrogating SPR glucometer that uses a spectrometer of 1 nm spectral resolution. The optical power measurement scheme that is employed in this work that requires no spectrometer, benefits the miniaturization of the sensing system.

To investigate the effects of ambient temperature variation on the glucometer, we house the sensor head in a chamber that controls its ambient temperature from −10 to 40 °C. Under such temperature variation, we observe that the standard deviation of glucose concentration is estimated as 12.1 mg/dL. This indicates that the addition of the further temperature correction function in the plasmonic fiber sensor would improve such a deviation problem, with the possibility of outperforming enzyme electrode based glucometers in temperature variation reliability.
